# The prognostic potential of fragmented CK18 serum levels in HCC patients reflecting disease progression and overall hepatocyte damage

**DOI:** 10.3389/fonc.2022.993705

**Published:** 2022-08-23

**Authors:** Akiko Eguchi, Motoh Iwasa, Yasuyuki Tamai, Minori Yamada, Koji Okuno, Ryuta Shigefuku, Kyoko Yoshikawa, Mina Tempaku, Koji Sakaguchi, Hideaki Tanaka, Kazushi Sugimoto, Yoshinao Kobayashi, Tetsuji Yamaguchi, Hayato Nakagawa

**Affiliations:** ^1^ Department of Gastroenterology and Hepatology, Graduate School of Medicine, Mie University, Tsu, Japan; ^2^ Bio-Reagent Material Development, Bio-Diagnostic Reagent Technology Center, Sysmex Corporation, Kobe, Japan; ^3^ Scientific Affairs, Sysmex Corporation, Kobe, Japan; ^4^ Center for Physical and Mental Health, Graduate School of Medicine, Mie University, Tsu, Japan; ^5^ Manufacturing Technology Development 1, Reagent Production, Sysmex Corporation, Kobe, Japan

**Keywords:** cytokeratin 18 (CK18), prognostic factor, hepatocellular carcinoma (HCC), liver cirrhosis, fCK18, fragmented cytokeratin 18, mortality

## Abstract

**Background:**

Fragmented cytokeratin 18 (fCK18) is released from damaged hepatocytes undergoing apoptosis and is recognized as a liver condition biomarker. We have developed a highly sensitive serum fCK18 CLEIA and reported that serum levels of this caspase-derived protein were significantly associated with hepatocyte ballooning, thus assisting in the accurate diagnosis of nonalcoholic steatohepatitis (NASH). We aim to investigate serum fCK18 levels in a variety of chronic liver diseases and to explore its potential as a prognostic marker of survival in hepatocellular carcinoma (HCC) patients.

**Methods:**

Serum fCK18 levels were measured using a highly sensitive CLEIA in 497 chronic liver disease patients (297 outpatients and 200 hospitalized with HCC).

**Results:**

In 497 chronic liver disease patients, serum fCK18 levels were significantly correlated with overall liver condition, including ALT, FIB-4 index and albumin-bilirubin (ALBI) score and were significantly increased in patients with HCC. In 200 HCC patients, serum fCK18 levels were significantly correlated with alpha-fetoprotein (AFP) and des-gamma-carboxy prothrombin (DCP), and were significantly associated with HCC stage, whereas FIB-4 index and ALBI score were not changed based on HCC stage. The Survival group had significantly lower levels of serum fCK18, AFP, DCP, FIB-4 index and ALBI score. A ROC analysis yield area under the curve (AUC) value of 0.728 for serum fCK18 is a significantly high value when compared to AUC measurements for other factors. Notably, AUROC values for serum fCK18 levels were constant in the short- and long-term by time-dependent ROC analysis for the prediction of HCC patient survival. HCC patients with serum fCK18 measured at < 1.15 ng/mL, AFP < 7.7 ng/mL, DCP < 133 mAU/mL, ALBI score < -2.97 or FIB-4 index < 6.4 had significantly longer rates of survival when compared to patients with values exceeding these thresholds. Serum fCK18 (HR, 3.5; *P* < 0.0001), DCP (HR, 3.2; *P* < 0.0001) and Barcelona Clinic Liver Cancer (BCLC) (HR, 2.4; *P* = 0.001) values were independent predictors of patient survival. [Conclusion] Serum fCK18 levels reflect overall liver function, the level of liver fibrosis and the progression of HCC, and are a potential predictor of survival in HCC patients.

## Introduction

Hepatocellular carcinoma (HCC) is the third leading cause of cancer-related deaths worldwide and one of the leading causes of death in patients with cirrhosis (1). Recommended surveillance guidelines for patients at high risk for HCC include abdominal imaging, using ultrasonography coupled with serum alpha-fetoprotein (AFP) measurement, followed by dynamic computed tomography (CT) or magnetic resonance imaging (MRI) (2–5). However, some patients with advanced HCC do not secrete AFP, moreover AFP can be elevated in conditions other than HCC, such as chronic hepatitis (6). Because of this, various biomarkers such as des-gamma-carboxy prothrombin (DCP) and AFP Lens culinaris agglutinin-reactive fraction of AFP (AFP-L3) have been developed and reported to be clinically useful (7, 8), however a prognostic marker with the ability to predict HCC patient survival has not been discovered.

Cytokeratins are major structural proteins found in epithelial cells that form a network of intermediate filaments in the cytoplasm. Cytokeratins consist of at least 20 unique gene products and are classified into two groups: type I (CK9-CK20) and type II (CK1-CK8) (9). Of particular importance to us in this manuscript is CK18, which is cleaved at various sites by activated caspases during apoptosis yielding several fragments, including 19 kD fragments that are released into the blood (10, 11). The process of apoptosis occurs as epithelial tumors progress, resulting in the elevation of serum CK18, and fragmented CK18 (fCK18) (12–15). However, few reports exist outlining the utility of serum fCK18 values with respect to HCC patients presenting with macrovascular invasion (16), therapy evaluation of transarterial chemoembolization (TACE) (17) or predictive outcomes of one-year survival rates after liver transplantation (18).

Recently, we have established a highly sensitive chemiluminescent enzyme immunoassay (CLEIA) that uses a new monoclonal antibody targeted against the caspase-derived 19 kD fragments of serum fCK18 (19). We have demonstrated that serum fCK18 levels were significantly associated with hepatocyte ballooning thus making them useful in diagnosing nonalcoholic steatohepatitis (NASH) (20). In this manuscript, we use this new highly sensitive CLEIA to investigate the association between serum fCK18 levels and markers of HCC, including HCC stage, and explore the predictive capacity of serum fCK18 with respect to HCC patient rate of survival.

## Materials and methods

### Human samples

The study protocol was approved by the Clinical Research Ethics Review Committee of Mie University Hospital. This study was performed retrospectively on stored samples from 2015 to 2016 and patients can opt out of their data being used. Written informed consent was obtained from all subjects at the time of blood sampling. Outpatients (n = 297) were recruited by their stage of chronic liver disease. Hospitalized HCC patients (n = 200) were also recruited. For HCC surgical resection, additional 34 HCC patients were recruited. Patients positive for hepatitis B surface antigen were diagnosed with hepatitis B virus (HBV) infection, whereas those positive for hepatitis C virus (HCV) RNA were diagnosed with HCV infection. Alcohol-associated liver disease (ALD) was defined as the presence of alcohol consumption > 60 g/day. NASH was diagnosed based on pathological findings in some of 200 hospitalized and 34 HCC liver resection patients and fatty liver without any other evident causes of chronic liver diseases (viral, autoimmune, genetic, etc.). HCC was diagnosed based on dynamic CT, MRI and/or pathological findings obtained during the clinical course. Barcelona Clinic Liver Cancer (BCLC) and tumor node metastasis (TNM) staging were determined based on criteria for TNM staging for HCC presented by the Liver Cancer Study Group of Japan and were used in the evaluation of tumor progression. Patients who had other malignancies within the past 3 years, severe hepatic failure (MELD score ≥ 30), uncontrollable infection, heart failure greater than the New York Heart Association–defined category of class II, human immunodeficiency virus (HIV) infection, pregnancy, or psychiatric problems were deemed to be unsuitable for clinical study. As a general rule, the follow-up examinations included routine physical examinations, and biochemical tests (1-3 monthly) and diagnostic imaging studies including ultrasonography, dynamic CT, or MRI (3-6 monthly). All treatments were performed following the Japanese practical guidelines for HCC as possible.

## Blood preparation

Clinical records including alanine aminotransferase (ALT), aspartate aminotransferase (AST), albumin (ALB), gamma-glutamyltransferase (γ-GT), total bilirubin (T-Bil), prothrombin time (PT), AFP and DCP were retrospectively evaluated. Blood samples were kept at -80 d until fCK18 measurement using the HISCL-5000 CLEIA system (Sysmex Corporation, Japan) (19) according to manufacturer’s instructions. The fibrosis index based on 4 factors (Fibrosis-4 [FIB-4]) (21) and albumin-bilirubin (ALBI) score (22) were calculated.

## Statistical analyses

All data are expressed as mean ± standard deviation, and categorical variables are shown as numbers of patients. Data were analyzed using Mann-Whitney U test in two groups, Kruskal-Wallis test for comparison of continuous variables and Wilcoxon test in the same HCC patients who received the surgical therapy. Correlation was determined using single regression analysis or Spearman rank-sum test. Receiver operator characteristic (ROC) curves and the corresponding area under the curve (AUC) were used to obtain cut-offs for the outcomes. The Youden index was applied to calculate the optimal cut-off point. We also conducted time-dependent ROC analysis for the prediction of HCC patient survival or death based on duration after HCC diagnosis. The cumulative survival rates were estimated with the Kaplan-Meier method and compared between groups by the log-rank test. Associations between predictor variables and overall survival were determined by the hazard ratio (HR) and a 95% confidence interval (CI) was calculated using Cox proportional hazards regression. The statistical analyses were performed using Prism 9 (GraphPad Software, Inc., CA, USA) for comparison of continuous variables and JMP version 9.0 (SAS Institute Japan, Inc., Tokyo) for univariate and multivariate Cox regression analyses. Differences were considered to be significant at *P* < 0.05.

## Result

### Serum fCK18 levels correlate with liver condition and are elevated in the patients with HCC

**Figure 1:**
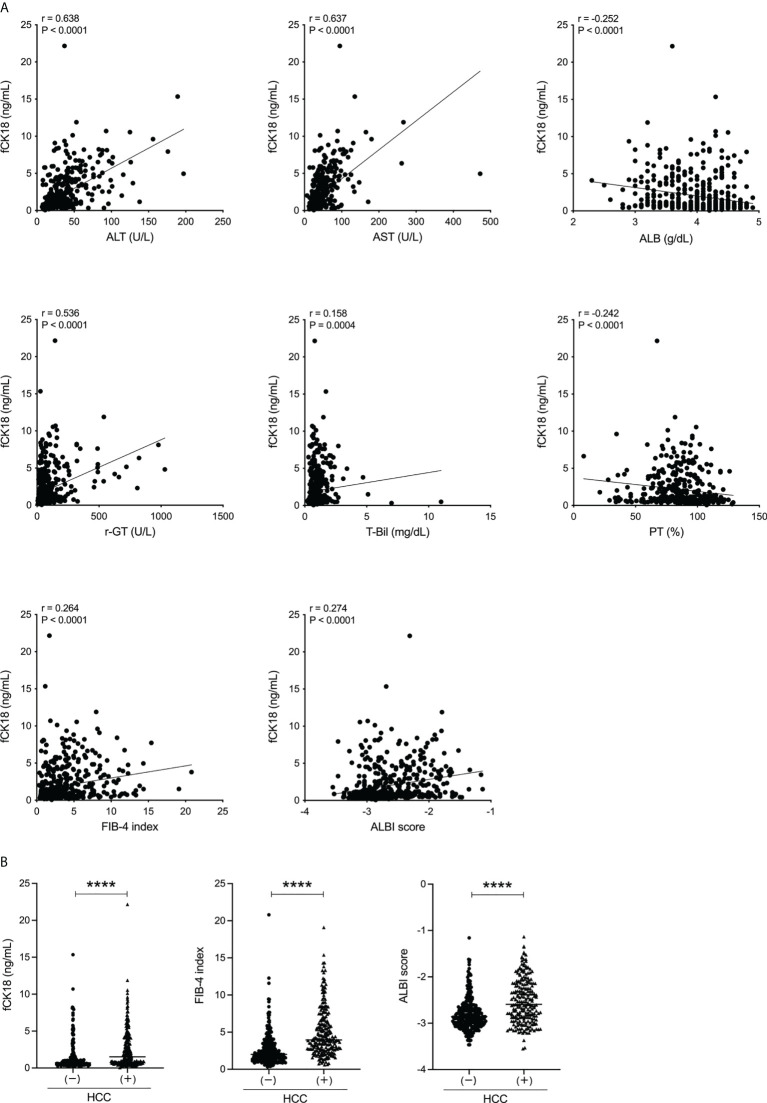
Serum fCK18 levels are correlated with liver function in human chronic liver disease and increased in HCC patients. **(A)** Correlation between serum fCK18 levels and ALT, AST, ALB, γ-GT, T-Bil, PT, FIB-4 index and ALBI score. **(B)** Serum fCK18 levels, FIB-4 index and ALBI score in patients with or without HCC. ****p<0.0001. ALT, alanine aminotransferase; AST, aspartate aminotransferase; ALB, albumin; γ-GT, gamma-glutamyltransferase; T-Bil, total bilirubin; PT, prothrombin time; FIB-4, fibrosis-4; ALBI, albumin-bilirubin; fCK18, fragmented cytokeratin 18.

We investigated whether serum fCK18 levels reflect overall liver condition in human chronic liver diseases of varying etiology. We recruited 497 patients (293 males, 204 females) with a mean age of 67.4 ± 12.4 years old. The cohort of study patients was admitted to our investigation based on a variety of causative agents: 54 HBV, 193 HCV, 116 nonalcoholic fatty liver disease (NAFLD)-NASH, 65 ALD, 27 autoimmune hepatitis, 29 primary biliary cholangitis, one (undefined) and 12 with no detectable liver disease (**Table 1**). We identified 229 (29 outpatients and 200 hospitalized patients) out of 497 patients who presented with HCC in addition to their underlying chronic liver disease (**Table 1**). Serum fCK18 levels were significantly correlated with ALT (*r* = 0.638; *P* < 0.0001), AST (*r* = 0.637; *P* < 0.0001), ALB (*r* = −0.252; *P* < 0.0001), γ-GT (*r* = 0.536; *P* < 0.0001), T-Bil (*r* = 0.158; *P* = 0.0004), PT (*r* = −0.242; *P* < 0.0001), FIB-4 index (*r* = 0.264; *P* < 0.0001) and ALBI score (*r* = 0.274; *P* < 0.0001) (**Figure 1A**), suggesting a predictive ability inherent in serum fCK18 levels for liver damage and fibrosis. To further investigate whether apoptosis in HCC can be detected by a highly sensitive CLEIA, we divided our patient cohort into two groups: with or without HCC. Serum fCK18 levels were significantly elevated in HCC patients compared to those without HCC (*P* < 0.0001) (**Figure 1B**). In our cohort, FIB-4 index and ALBI score measurements were shown to be significantly increased in patients with HCC (*P* < 0.0001) (**Figure 1B**).

**Table 1:** All patient characterization.

	N = 497
Age, years	67.4 ± 12.4
Gender, male/female	293/204
Etiology (HBV/HCV/NAFLD-NASH/alcohol/AIH/PBC/others/no liver disease)	54/193/116/65/27/29/1/12
HCC (-/+)	268/229 (229: 29 outpatients and 200 hospitalized patients)
ALB (g/dL)	4.0 ± 0.5
ALT (IU/L)	31.4 ± 25.7
AST (IU/L)	40.9 ± 34.9
**γ**-GT (IU/L)	75.8 ± 120.6
T-Bil (mg/dL)	1.0 ± 0.7
PT (%)	87.2 ± 19.9
FIB-4 index	3.7 ± 3.0
ALBI score	-2.7 ± 0.4

Values are mean ± SD.

ALB, albumin; ALT, alanine aminotransferase; AST, aspartate aminotransferase; γ-GT, gamma-glutamyltransferase; T-Bil, total bilirubin; PT, prothrombin time; FIB-4, Fibrosis-4; ALBI, albumin-bilirubin; NAFLD, nonalcoholic fatty liver disease; NASH, nonalcoholic steatohepatitis; AIH, autoimmune hepatitis; PBC, primary biliary cholangitis.

### Serum fCK18 levels significantly increase in HCC

**Figure 2:**
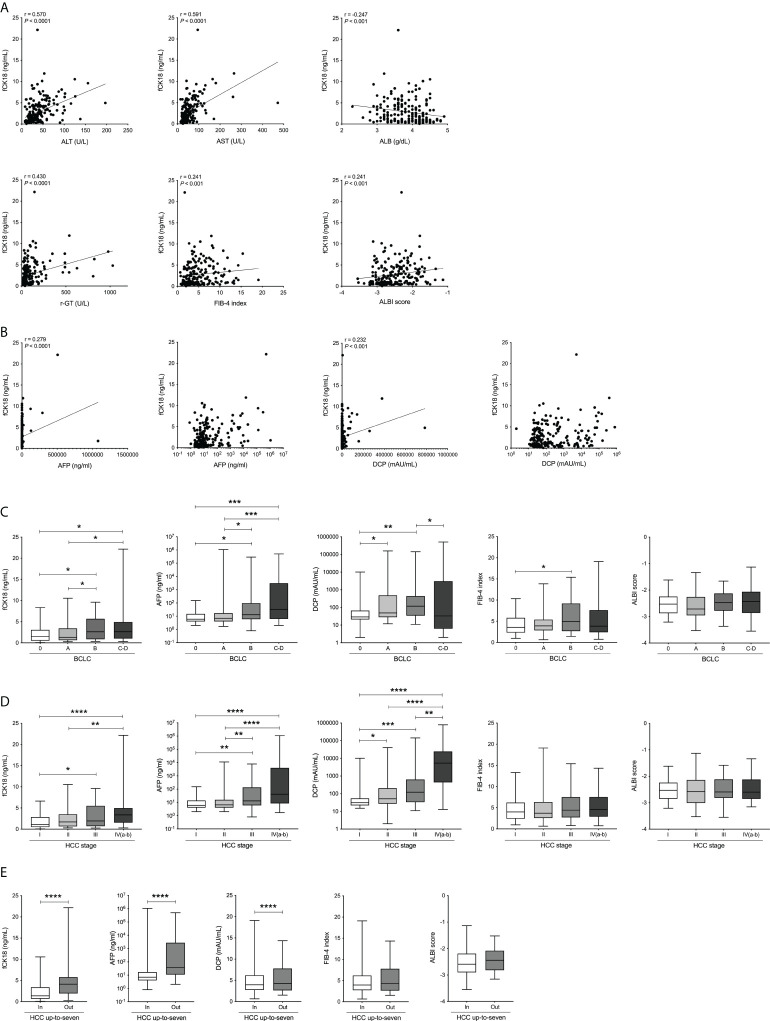
Serum fCK18 levels are associated with HCC markers and BCLC stage. **(A)** Correlation between serum fCK18 levels and ALT, AST, ALB, γ-GT, T-Bil, ALBI score and FIB-4 index. **(B)** Correlation between serum fCK18 levels and HCC markers, such as AFP and DCP, with linear and log axis **(C–E)** Serum fCK18 levels, FIB-4 index and ALBI score in **(C)** BCLC stage, **(D)** HCC stage or **(E)** in or out of HCC up-to-seven. Values are boxed with minimum/maximum. ****p<0.0001, ***p<0.001, **p<0.01, *p<0.05. ALB, albumin; ALT, alanine aminotransferase; AST, aspartate aminotransferase; γ-GT, gamma-glutamyltransferase; T-Bil, total bilirubin; ALBI, albumin-bilirubin; FIB-4, fibrosis-4; fCK18, fragmented cytokeratin 18; AFP, alpha-fetoprotein; DCP, des-gamma-carboxy prothrombin; BCLC, Barcelona Clinic Liver Cancer; HCC, hepatocellular carcinoma.

To explore the association between serum fCK18 and HCC stage, we focused on 200 hospitalized HCC patients on whom detailed tumor information was available. This HCC cohort included 154 males and 46 females with a mean age of 71.3 ± 9.8 years old. The cohort of study patients was admitted to our investigation based on a variety of causative agents: 20 HBV, 102 HCV, 43 NAFLD-NASH, 28 ALD and seven others (**Table 2**). Serum fCK18 levels were significantly correlated with ALT (*r* = 0.570; *P* < 0.0001), AST (*r* = 0.591; *P* < 0.0001), ALB (*r* = −0.247; *P* < 0.001), γ-GT (*r* = 0.430; *P* < 0.0001), FIB-4 index (*r* = 0.241; *P* < 0.001) and ALBI score (*r* = 0.241; *P* < 0.001) (**Figure 2A**), indicating a predictive ability inherent in serum fCK18 levels for liver damage in HCC. Moreover, serum fCK18 levels were significantly correlated with several blood tumor markers, namely AFP (*r* = 0.279; *P* < 0.0001) and DCP (*r* = 0.232; *P* < 0.001) (**Figure 2B**), although the correlation coefficient is low. We observed gradually increasing serum fCK18 levels corresponding to BCLC stage (BCLC 0 versus BCLC B or C-D, BCLC A versus BCLC B or C-D; *P* < 0.05) (**Figure 2C**) and TNM staging of HCC (HCC stage) (stage I versus stage III; *P* < 0.05, stage I versus stage IVa-b; *P* < 0.0001, stage II versus stage IVa-b; *P* < 0.01) (**Figure 2D**). In addition, AFP and DCP were gradually increased corresponding to BCLC stage (AFP: BCLC 0 or A versus BCLC C-D; *P* < 0.001, BCLC 0 or A versus BCLC B; *P* < 0.05) (DCP: BCLC 0 versus BCLC B; *P* < 0.01, BCLC 0 versus BCLC A or BCLC B versus BCLC C-D; *P* < 0.05) (**Figure 2C**) and HCC stage (AFP: stage I or II versus stage IVa-b; *P* < 0.0001, stage I or II versus stage III; *P* < 0.01) (DCP: stage I or II versus stage IVa-b; *P* < 0.0001, stage I versus stage III; *P* < 0.001, stage III versus stage IVa-b; *P* < 0.01, stage I versus stage II; *P* < 0.05) (**Figure 2D**). In contrast, FIB-4 index and ALBI score measurements were not changed based on BCLC and HCC stages, although the FIB-4 index value was increased in BCLC B when compared to BCLC 0 (*P* < 0.05) (**Figures 2C, D**). Serum fCK18 levels, as well as AFP and DCP, were significantly elevated in the HCC up-to-seven group (largest tumor diameter [in cm] + tumor number > 7) (23) (*P* < 0.0001), while FIB-4 index and ALBI score was the same between groups in or out of the HCC up-to-seven criteria (**Figure 2E**). These results indicate that serum fCK18 levels are associated with HCC progression and may reflect HCC-driven apoptosis in addition to hepatocellular damage.

**Table 2:** HCC Patient characterization.

	n=200
Age, years	71.3 ± 9.8
Gender, male/female	154/46
Etiology, HBV/HCV/NASH/alcohol/others	20/102/43/28/7
BCLC (0/A/B/C/D)	39/76/41/41/3
ALB (g/dL)	3.9 ± 0.5
ALT (IU/L)	38.2 ± 28.7
AST (IU/L)	54.0 ± 47.3
**γ**-GT (IU/L)	103.9 ± 155.7
T-Bil (mg/dL)	1.0 ± 0.6
FIB-4 index	5.0 ± 3.3
ALBI score	-2.5 ± 0.5

Values are mean ± SD.

ALB, albumin; ALT, alanine aminotransferase; AST, aspartate aminotransferase; γ-GT, gamma-glutamyltransferase; T-Bil, total bilirubin; FIB-4, Fibrosis-4; ALBI, albumin-bilirubin; NASH, nonalcoholic steatohepatitis; BCLC, Barcelona Clinic Liver Cancer.

### Serum fCK18 levels are significantly increased in deceased HCC patients and have an impact on short-term to long-term survival

**Figure 3:**
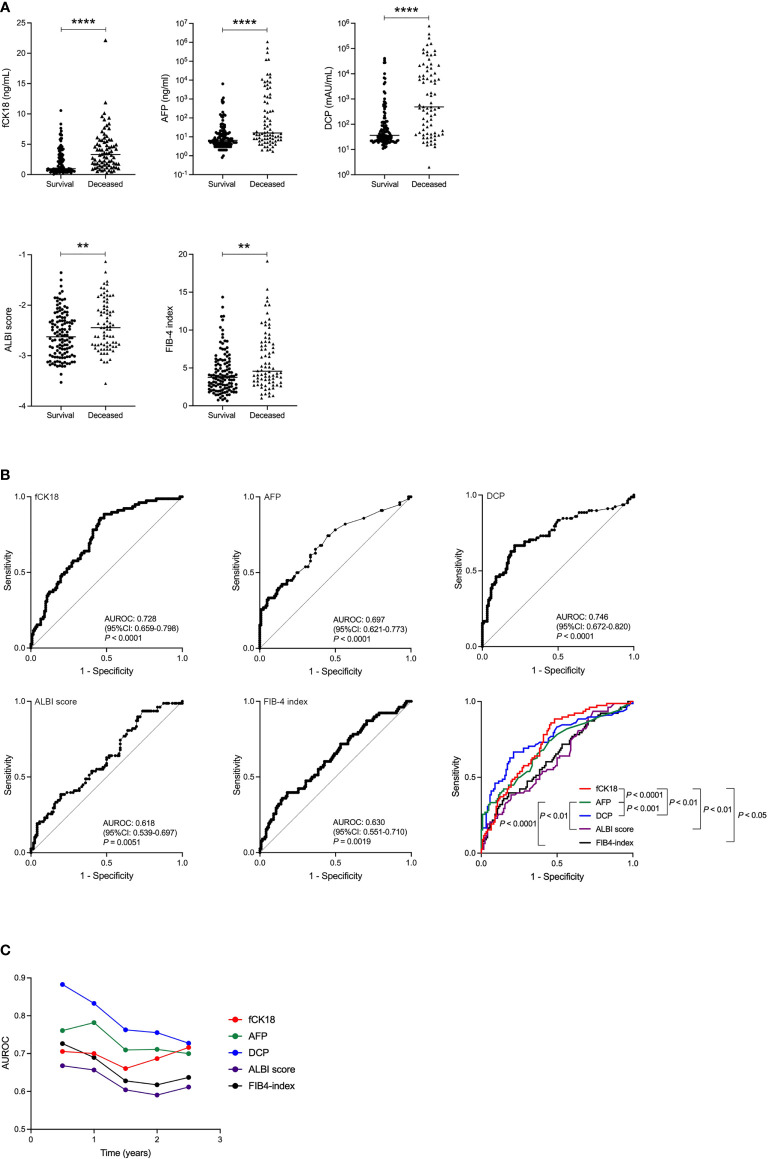
Survival rate improved in HCC patients indicated by serum fCK18 levels. **(A)** Serum fCK18, AFP and DCP levels, as well as ALBI score and FIB-4 index, in Survival and Deceased groups in HCC cohort. **(B)** ROC curve of serum fCK18, AFP and DCP levels, as well as ALBI score and FIB-4 index. ROC curve of all five factors. **(C)** Plots of 0.5 year AUROC for serum fCK18 levels, AFP, DCP, ALBI score and FIB-4 index. ****p<0.0001, **p<0.01. fCK18, fragmented cytokeratin 18; AFP, alpha-fetoprotein; DCP, des-gamma-carboxy prothrombin; ALBI, albumin-bilirubin; FIB-4,fibrosis-4; AUROC, area under receiver operating characteristic curve.

We examined the prognosis of 200 HCC patients and found that 78 patients died (henceforth called Deceased) within the follow-up period of 995.5 ± 409.5 days. Serum fCK18 levels were significantly increased in the Deceased group compared to the Survival group (*P* < 0.0001) (**Figure 3A**). Several blood tumor markers, AFP and DCP, and liver condition markers, ALBI score and FIB-4 index, were also significantly increased in the Deceased group compared to the Survival group (AFP and DCP; *P* < 0.0001, ALBI score and FIB-4 index; *P* < 0.01) (**Figure 3A**). ROC analyses yielded AUC values of 0.728 (95% CI: 0.659-0.798; *P* < 0.0001) for serum fCK18 levels (**Figure 3B**), whereas AUC values of 0.697 (95% CI: 0.621-0.773; *P* < 0.0001), 0.746 (95% CI: 0.672-0.820; *P* < 0.0001), 0.618 (95% CI: 0.539-0.697; *P* = 0.0051) and 0.630 (95% CI: 0.551-0.710; *P* = 0.0019) were observed for AFP, DCP, ALBI score and FIB-4 index, respectively (**Figure 3B**). In ROC analysis, AUC values for serum fCK18 were significantly higher than those of AFP (*P* < 0.0001), ALBI score (*P* < 0.01) and FIB-4 index (*P* < 0.05) (**Figure 3B**). We further conducted time-dependent ROC analysis for the prediction of HCC patient survival, or death, based on the duration of time post HCC diagnosis. AUROC values for HCC markers (AFP and DCP) were highest in the short-term, but decreased months after diagnosis (**Figure 3C**). In contrast, AUROC values for ALBI score and FIB-4 index were lower in the short-term and long-term (**Figure 3C**). Notably, AUROC values for serum fCK18 levels were constant in the short-term and long-term (**Figure 3C**).

### Serum fCK18 levels are a prognostic factor for survival in HCC

**Figure 4:**
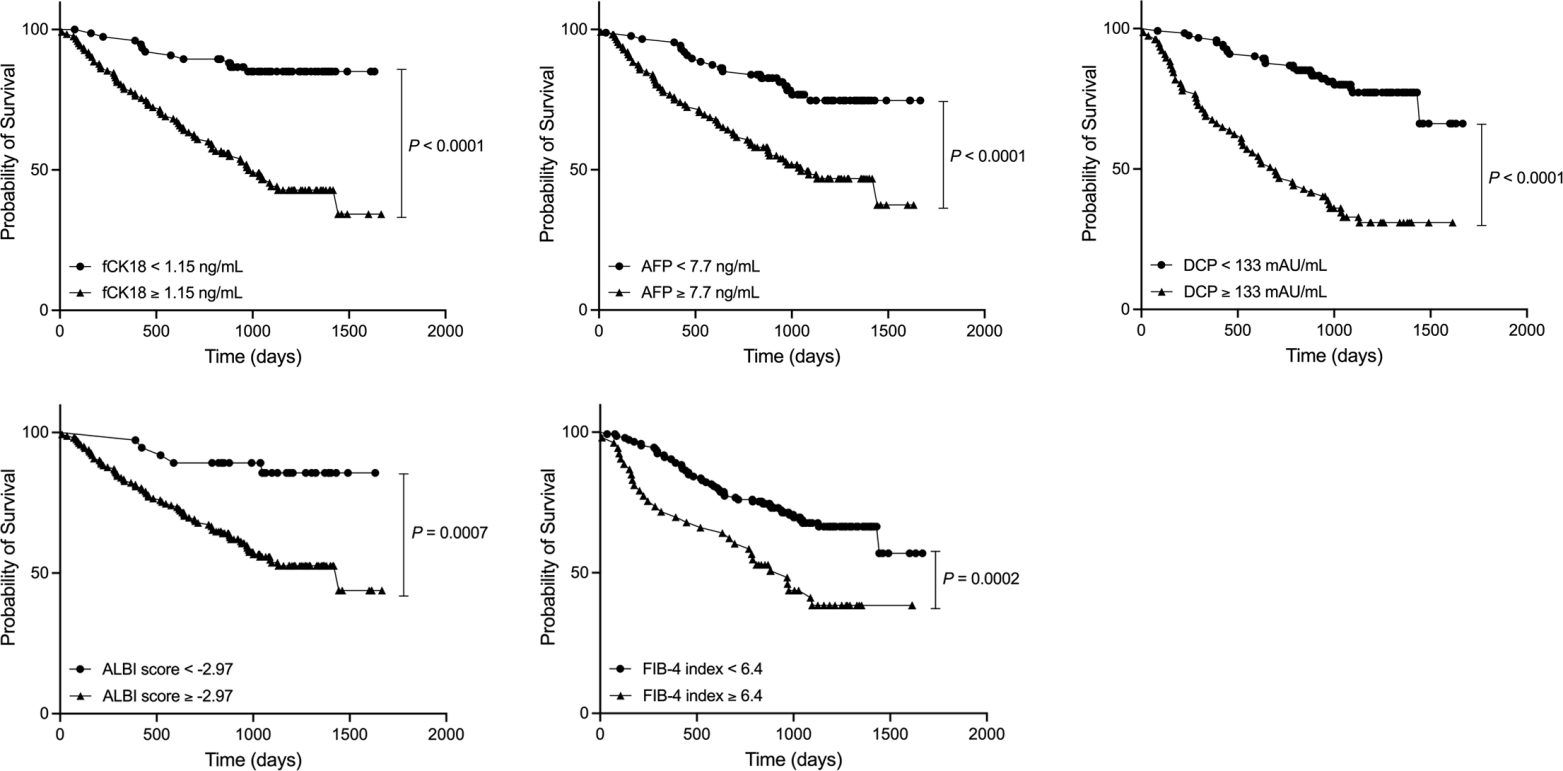
HCC patients with low serum fCK18 levels have longer rate of survival. Survival curve in HCC patients with serum fCK18, AFP and DCP levels, as well as ALBI score and FIB-4 index. fCK18, fragmented cytokeratin 18; AFP, alpha-fetoprotein; DCP, des-gamma-carboxy prothrombin; ALBI, albumin-bilirubin; FIB-4,fibrosis-4.

We calculated the cut-off value of serum fCK18 levels to be 1.15 ng/mL, AFP levels to be 7.7 ng/mL, DCP levels to be 133 mAU/mL, ALBI sore to be -2.97 and FIB-4 index to be 6.4. The survival rate was significantly decreased in patients with serum fCK18 levels ≥ 1.15 ng/mL compared to patients with fCK18 < 1.15 ng/mL (*P* < 0.0001) (**Figure 4**) and similar observations were made in patients with AFP ≥ 7.7 ng/mL (*P* < 0.0001), DCP ≥ 133 mAU/mL (*P* < 0.0001), ALBI score ≥ -2.97 and FIB-4 index ≥ 6.4 (**Figure 4**). To determine the significant factors driving survival in HCC, we used univariate Cox regression analysis and included gender, age, serum fCK18 levels, AFP, DCP, BCLC, ALBI score and FIB-4 index. It was determined that serum fCK18 levels (HR, 5.0; *P* < 0.00001), AFP (HR, 2.8; *P* < 0.0001), DCP (HR, 4.7; *P* < 0.0001), BCLC (HR, 4.4; *P* < 0.0001), ALBI score (HR, 4.2; *P* = 0.0001) and FIB-4 index (HR, 2.3; *P* = 0.0006) were independent predictors of survival (**Table 3**). Moreover, we used multivariate Cox regression analyses to assess serum fCK18 levels (HR, 3.5; *P* < 0.0001), DCP (HR, 3.2; *P* < 0.0001) and BCLC (HR, 2.4; *P* = 0.0011) as independent predictors of survival, but excluded AFP, ALBI score and FIB-4 index values (**Table 3**). These results reveal serum fCK18 levels to be a prognostic factor in HCC.

**Table 3:** Univariate and multivariate Cox regression analysis for predictors of survival in HCC patients.

	Univariate			Multivariate		
Variables	HR	95% CI	*P* Value	HR	95% CI	*P* Value
Gender Male	0.872	0.530-1.502	0.6095			
Age	1.326	0.843-2.123	0.2248			
fCK18 > 1.15	4.976	2.741-9.960	<0.0001	3.490	1.822-7.281	<0.0001
AFP > 7.7	2.765	1.693-4.710	<0.0001	1.343	0.658-2.046	0.6438
DCP > 133	4.671	2.941-7.603	<0.0001	3.244	1.921-5.570	<0.0001
BCLC > 0 and A	4.432	2.762-7.310	<0.0001	2.393	1.409-4.164	0.0011
ALBI score > -2.97	4.222	1.887-12.037	0.0001	2.084	0.882-6.134	0.0989
FIB-4 index >6.4	2.285	1.438-3.581	0.0006	1.550	0.950-2.503	0.0790

HCC, hepatocellular carcinoma; fCK18, fragment cytokeratin 18; AFP, alpha-fetoprotein; DCP, des-gamma-carboxy prothrombin; BCLC, Barcelona Clinic Liver Cancer; ALBI, albumin-bilirubin; FIB-4, Fibrosis-4; HR, hazard ratio; CI, confidence interval.

### Changes in serum fCK18 levels after surgical therapy for HCC

**Figure 5:**
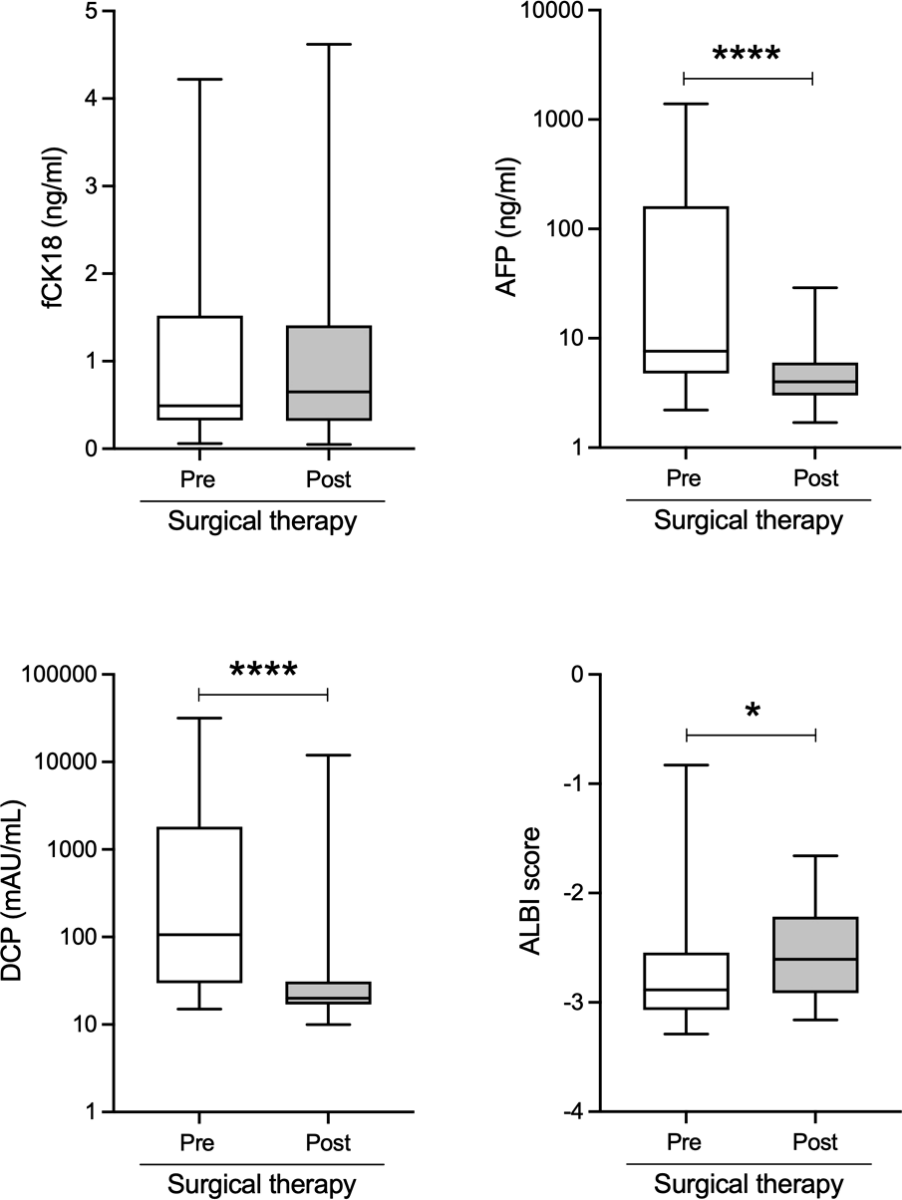
Serum fCK18 levels are not changed by the surgical therapy in HCC patients. Changes of serum fCK18, AFP and DCP levels, as well as ALBI score in HCC patients who received surgical resection. fCK18, fragmented cytokeratin 18; AFP, alpha-fetoprotein; DCP, des-gamma-carboxy prothrombin; ALBI, albumin-bilirubin. * p<0.05, **** p<0.0001.

To explore whether serum fCK18 levels have a potential for HCC marker, we measured serum fCK18 levels in 34 HCC patients who received surgical resection. This surgical therapy cohort included 29 males and five females with a mean age of 70.5 ± 10.1 years old, all Child-Pugh A class and BCLC 28 A/one B/five C within the follow-up period of 106.9 ± 120.4 days. The causative agents of liver disease were follows: seven HBV, seven HCV, six NASH, 13 ALD and one others. In this cohort, six HCC stage I, 21 HCC stage II, four HCC stage III and three HCC stage IV with 0.97 (0.06-4.2) ng/mL of serum fCK18 levels as a mean, suggested that majority of 34 HCC patients would not have high serum fCK18 levels due to early HCC stage. HCC markers, AFP and DCP, were significantly decreased and ALBI score were improved in HCC patients who received the surgical therapy (**Figure 5**). In contrast, serum fCK18 levels were not changed by the surgical therapy (**Figure 5**), suggesting that serum fCK18 levels were not useful to determine therapy effect.

## Discussion

Our study demonstrates that serum fCK18 levels are significantly increased in patients with HCC. Serum fCK18 levels are also associated with HCC stage and may be useful in predicting HCC patient survival, making this manuscript the first to outline a biomarker with this ability. Significant increases in serum fCK18 levels have been associated with hepatocyte damage, particularly apoptosis, and well-recognized in NASH (24). However, it is the unfortunate truth that fCK18 measurement cannot be applied clinically due to widely variable cut-off values and no correlation with hepatocyte ballooning under the current enzyme-linked immunosorbent assay platform (24). Notably, our highly sensitive CLEIA using new antibodies generated against fCK18 or CK18 can assess hepatocyte ballooning and distinguish fatty liver patients from healthy individuals (19, 20). Our highly sensitive CLEIA also enables the detection of HCC-related apoptosis resulting in the ability to predict overall survival in HCC patients within this study.

DCP and AFP are widely used surveillance markers for HCC, although some HCC patients present with low DCP or AFP levels (25), and DCP can be affected by the administration of warfarin and vitamin K antagonists (26). In contrast, fCK18 values reflect hepatocyte apoptosis throughout the entire liver, including damaged hepatocytes and HCC tissue. Indeed, serum fCK18 levels were significantly increased a few days after successful TACE treatment due to HCC apoptosis thus highlighting the usefulness of serum fCK18 levels as a measure of therapy efficacy (17). In this study, we used a cohort in which FIB-4 index and ALBI scores were not different based on HCC stage. We demonstrated that serum fCK18 levels were significantly elevated mirroring HCC progression, as assessed using BCLC and HCC stages and the up-to-seven criteria, suggesting that HCC itself releases fCK18 *via* apoptosis as a function of HCC progression. However, serum fCK18 levels were not changed by the liver resection in HCC patients, meaning that serum fCK18 levels are useful only for advanced HCC and prognostic purposes. We guess that the majority of 34 HCC patients who received the liver resection had an early HCC stage, thus serum fCK18 levels may not be high enough to explore the changed of serum fCK18 levels by the liver resection. We need further study to investigate the changes of serum fCK18 levels using a late HCC stage of patients. In general, tumor stage, ALBI score and nutritional status are well-known prognostic factors used in the assessment of HCC patients (27). Therefore, keeping favorable liver function/condition is essential in order to continue HCC treatments, and close monitoring of HCC progression and overall liver condition is required in the proper management of HCC patients. The present study clearly showed that all factors, including serum fCK18 levels, ALBI score, FIB-4 index, AFP and DCP were significant survival rate prognostic markers in patients with HCC. Time-dependent ROC analyses highlighted that serum fCK18 levels had a consistent impact on short- to long-term survival of HCC patients, while AFP and DCP had a strong impact on the short-term survival of patients with HCC. In this study, we showed the potential of serum fCK18 levels, in both HCC and residual functional liver, to provide a predictive capacity in gauging overall HCC patient survival.

This study is limited insofar as it is retrospective and all samples were collected from a single center. We need to validate these results using a multi-center study approach.

In conclusion, serum fCK18 levels correlate with liver function, liver fibrosis and the progression of HCC in patients spanning a variety of chronic liver diseases, which promotes the use of this measure as a means to predict survival in HCC patients.

## Data availability statement

The data analyzed in this study is subject to the following licenses/restrictions. Patient datasets are not available on the public. Requests to access these datasets should be directed to akieguchi@med.mie-u.ac.jp.

## Ethics statement

The studies involving human participants were reviewed and approved by the Clinical Research Ethics Review Committee of Mie University Hospital. Written informed consent for participation was not required for this study in accordance with the national legislation and the institutional requirements.

## Author contributions

AE, MI, YT and HN contributed to conception and design of the study. YT, MY, KO, RS, KY, MT, KSa, HT, and KSu organized the database. AE, YT and YK performed the statistical analysis. AE wrote the first draft of the manuscript. MI wrote sections of the manuscript. All authors contributed to manuscript revision, read, and approved the submitted version.

## Funding

This research was supported by JSPS KAKENHI Grant Number 21H02892, and AMED under Grant Number JP21fk0210090 and JP22fk0210115.

## Conflict of interest

Author's MY, KO, KS and TY were employed by Sysmex Corporation.

The remaining authors declare that the research was conducted in the absence of any commercial or financial relationships that could be construed as a potential conflict of interest.

## Publisher’s note

All claims expressed in this article are solely those of the authors and do not necessarily represent those of their affiliated organizations, or those of the publisher, the editors and the reviewers. Any product that may be evaluated in this article, or claim that may be made by its manufacturer, is not guaranteed or endorsed by the publisher.

## Abbreviations

AFP, alpha-fetoprotein; ALB, albumin; ALBI, albumin-bilirubin; ALT, alanine aminotransferase; ALD, alcohol-associated liver disease; AST, aspartate aminotransferase; AUC, area under the curve; BCLC, Barcelona Clinic Liver Cancer; CK18, cytokeratin 18; CT, computed tomography; DCP, des-gamma-carboxy prothrombin; FIB-4, Fibrosis-4; fCK18, fragmented cytokeratin 18; γ-GT, gamma-glutamyltransferase; HBV, hepatitis B virus; HCC, hepatocellular carcinoma; HCV, hepatitis C virus; HR, hazard ratio; NAFLD, nonalcoholic fatty liver disease; NASH, nonalcoholic steatohepatitis; MRI, magnetic resonance imaging; PT, prothrombin time; ROC, receiver operating characteristic curve; T-Bil, total bilirubin.
